# Postoperative sore throat: prophylaxis and treatment

**DOI:** 10.3389/fphar.2023.1284071

**Published:** 2023-11-23

**Authors:** Elvio Mazzotta, Suren Soghomonyan, Ling-Qun Hu

**Affiliations:** ^1^ The Ohio State University, Columbus, United States; ^2^ Wexner Medical Center, The Ohio State University, Columbus, OH, United States

**Keywords:** postoperative sore throat, postoperative complications, intratracheal intubation, endotracheal tube, anesthetic complications

## Abstract

Postoperative sore throat (POST) is one of the most reported complications after general anesthesia with an incidence of as high as 60% which may impact patient satisfaction and increase the cost of treatment. The aim of this review is to summarize the currently accepted approaches and new trends intended to reduce the risk and increase the treatment efficacy of POST. Difficult intubation, traumatic intubation, and several other factors contribute to the development of POST. Endotracheal intubation using a stylet-loaded tube exerts excessive pressure on the anterior tracheal wall predisposing to mucosal trauma and contributing to development of POST. Pharmacological interventions are aimed at prevention, amelioration of symptoms, and treatment of POST. Medications suggested for this purpose include corticosteroids, topical sprays and creams, non-steroidal anti-inflammatory drugs (NSAID), and N-methyl-D-aspartate (NMDA) receptor antagonists. The use of video-laryngoscopes (VL) for endotracheal intubation improves the glottic view and increases the success rates with less force required to ensure adequate laryngoscopic view. Nevertheless, despite advances in laryngoscopic devices, the incidence of POST remains high. A novel intubation technique with endotracheal tube (ETT) rotation 180 degrees (ETT 180°) has been suggested to overcome stylet related injury and, possibly, decrease the POST. To date, no clinical trials have been conducted to test the efficacy of ETT 180° in reducing the incidence of POST. Undoubtedly, the suggested method deserves further investigation to determine its role in patient care.

## Introduction

POST is a well-documented complication after tracheal intubation with an incidence of as high as 60% with a significant negative impact on patients’ recovery and satisfaction (Macario et al., 1999; McHardy and Chung, 1999; Scuderi, 2003; Boghdadly et al., 2016). The term POST is not well defined and usually describes a wide variety of conditions including pharyngitis, laryngitis, tracheitis, cough, hoarseness or dysphagia manifesting in the early postoperative period (Higgins et al., 2002; Boghdadly et al., 2016). Several risk factors for its development have been reported including female sex, younger age, pre-existing lung disease, prolonged duration of anesthesia, size of tracheal tube, double lumen ETT, presence of a blood-stained ETT on extubation, and high ETT cuff pressure exceeding 20 cm H_2_O (Boghdadly et al., 2016; Mitobe et al., 2022).

The etiology of POST is complex and multiple mechanisms may contribute including airway trauma and irritation with mucosal injury and inflammation, prolonged ischemia of the mucosa caused by mechanical pressure, regurgitation of the gastric contents, placement of a gastric tube, etc. (McHardy and Chung, 1999; Roffey et al., 2003; Scuderi, 2003; Liu et al., 2010) Among many factors, mechanical injury of the airway mucosa caused by forceful laryngoscopy and the use of a stylet loaded ETT are considered the main culprits (Komasawa et al., 2017). The available evidence suggests that the mechanical impact on the anterior tracheal wall resulting from the removal of a stylet (Boghdadly et al., 2016; Kusunoki et al., 2016) during endotracheal intubation may be a key factor contributing to POST (Chou and Wu, 2001). Furthermore, the extraction force during stylet removal seems to play a significant role.

A prospective study by Kusunoki et al. (2016) has shown that increased extraction force during stylet removal exceeding 10.3 Newtons is associated with high incidence of POST).

Despite the risks associated with stylets, including POST, their use to facilitate the endotracheal intubation is unavoidable in many cases, and new modifications of intubation techniques aimed at reducing the associated complications should be encouraged (Komasawa et al., 2017). Efforts have been made to decrease the incidence and severity of POST by either modifying the intubation technique or using topical and systemic pharmacotherapy. Nevertheless, POST remains a common complication of anesthesia requiring attention (Boghdadly et al., 2016). This review focuses on current practices in medical treatment of POST as well as a newly suggested modification of endotracheal intubation which has the potential to reduce the incidence of POST.

## Pharmacological interventions

In the context of multimodal analgesia, several pharmacological interventions have been tested to reduce the incidence of POST and increase the quality of recovery. In this section we will briefly describe the most updated recommendations for POST prevention.

### Steroids and NSAIDs

The use of glucocorticoids and NSAIDs is justified in the management of POST considering the role of inflammation in pathogenesis of POST*.* An updated meta-analysis on the efficacy of dexamethasone in reducing the incidence of POST showed that dexamethasone 0.2 mg/kg significantly decreased the incidence of POST, whereas dexamethasone at a dose of 0.1 mg/kg was not effective (Jiang et al., 2018). Topical steroids represent an additional therapeutic option. According to literature reports, topical corticosteroids, when applied to the tracheal mucosa, reduce the incidence of POST when compared with non-analgesic control drug, 95% confidence interval (CI) 0.39 (0.32–0.49) (18 trials including 1506 patients) (Kuriyama et al., 2018a).

NSAIDs are very effective in reducing postoperative pain. Benzydamine hydrochloride, a topical NSAID with analgesic and anti-edema properties, has been proposed as a treatment option for prevention of POST. A meta-analysis by Kuriyama et al. assessed the efficacy of Benzydamine. In the study involving 1842 patients, Benzydamine treatment was associated with a significant decrease in POST with a risk ratio (RR) 0.31, 95% CI 0.20–0.47, and the number needed to prevent of 6 (95% CI 5–8), indicating a significant relevant prophylactic effect (Kuriyama et al., 2018b). Subsequently, another RCT tested flurbiprofen lozenge as a preoperative intervention to address POST and dysphagia associated with the use of laryngeal mask airway (LMA). The study found that the use of flurbiprofen lozenge at dose 8.75 mg effectively reduced the severity, but not the incidence of early POST (Uztüre et al., 2014). Evidence assessing the efficacy of intravenous NSAIDs on POST is currently limited, with only one randomized controlled trial (RCT) testing intravenous Diclofenac that showed no protective effect (Thanga et al., 2013).

### Lidocaine

According to a 2015 Cochrane meta-analysis of 1940 patients, topical (intracuff lidocaine, lidocaine jelly, and lidocaine spray) and systemic lidocaine appeared to reduce the incidence of POST (16 studies, 1774 participants, RR = 0.64, 95% CI 0.48–0.85). However, the effect was no longer significant, when only high-quality trials were included (eight studies, 814 participants; RR 0.71, 95% CI 0.47–1.09) (Thanga et al., 2013). Similarly, Li et al. (2020) conducted a recent meta-analysis assessing the efficacy of topical and systemic lidocaine. Their study showed that intracuff and intravenous lidocaine (dose 1.5 mg/kg) effectively reduced the risk of POST at 1 h and 24 h, but lidocaine jelly and spray were not. Despite these positive results, the level of heterogenicity among studies was high and results should be interpreted with caution (Li et al., 2020).

### NMDA receptor antagonists

Magnesium and ketamine, two NMDA receptor antagonists, are commonly used in anesthesia for their antinociceptive and anti-inflammatory properties and have been studied for their potential to reduce POST. Several meta-analyses looked at the effectiveness of topical magnesium sulfate prior to surgery in preventing POST. One study used the number needed to treat (NNT) to assess the efficacy. Magnesium was administered as gargles (20 mg/kg), lozenges (100 mg) or nebulization (225–500 mg) 15–30 min before surgery in preoperative area. According to the authors, the NNT equaled 5.76 patients to be treated to prevent 1 event of POST (Singh et al., 2019). Similarly, a recent network meta-analysis reported that topical magnesium effectively prevented the POST 24 h after endotracheal intubation (odds ratio 0.10, and 95% credible interval 0.03–0.26) (Singh et al., 2020). Similarly, a meta-analysis of 41 RCT involving over 3000 patients showed that topical ketamine, regardless of the administration method (gargle 20–50 mg, nebulized 0.5–1.5 mg/kg or lubrication of ETT 50 mg) was associated with lower incidence of POST in the first 24 h (RR 0.45; 95% CI 0.37-0.54; *p* < 0.001) (Kuriyama et al., 2020). On the contrary, the use of IV ketamine 0.5 mg/kg bolus follow by a low maintenance infusion in a RCT, did not result in a significant reduction in the incidence of POST (Park et al., 2010).

### Other drugs

Liquorice, also spelled as “licorice”, is derived from the root of Glycyrrhiza gabra and has long history of use in medicine due to its various properties including anti-inflammatory and antitussive effects (Fiore et al., 2005). An early RCT by Arwal et al. showed a reduction in the incidence of POST both at rest and on swallowing after a gargle of 0.5 gr of liquorice made in 30 mL of water (Agarwal et al., 2009). Similarly, a recent meta-analysis of more than 70 trials revealed a reduction of POST with the usage of topical liquorice before induction of anesthesia (Boghdadly et al., 2016).

### Non-pharmacological interventions and a novel intubation approach

#### ETT sizing

Selecting the appropriate endotracheal tube (ETT) size is crucial in minimizing the risk of sore throat and ensuring effective airway management during intubation. Generally, an internal diameter (ID) (6.0–7.5 mm) ETTs are generally suitable for females, whereas 7.0–8.0 mm ID ETTs are suitable for males (Butterworth, 2013). Studies have shown that female patient under general anesthesia with smaller size of ETT (6.0 mm) were associated with a lower incidence of POST (Hu et al., 2013; Jaensson et al., 2014).

#### Tracheal cuff pressure

Higher cuff pressures in endotracheal tubes (ETTs) and supraglottic airway (SGA) devices can indeed be associated with an increased incidence of sore throat. This is due to the potential for mucosal trauma and pressure-related injuries when the cuff pressure is excessively high (Ansari et al., 2014). To mitigate the risk of sore throat associated with high cuff pressures, monitoring cuff pressures regularly and adjust them as needed to maintain an appropriate range has been suggested. In a prospective randomized control trial on patients undergoing thyroidectomy, the authors showed that monitoring and maintaining cuff pressure at 25 cm H2O was associated with less incidence and severity of POST at 2 (61% vs. 86%; *p* = 0.008) and 24 h postoperatively (43% vs. 66%; *p* = 0.032) (Ryu et al., 2013).

Similarly, another prospective study on patients undergoing maxillofacial surgery, offers further support for the practice of monitoring and adjusting cuff pressure intraoperatively to reduce the incidence of POST (Ansari et al., 2014).

#### Video laryngoscopes

VL have been popularized due to enhanced glottic view and high rate of successful intubations (Hoshijima et al., 2018). Their introduction shifted the paradigm in airway management with enhanced intubation rates and most likely may soon become a standard of care (Najafi et al., 2014; Prekker et al., 2023). A trade-off for the superior glottic view with VL, the ETT must be curved to a considerably more acute angle to enable insertion and match the laryngoscope’s angle of view. The manufacturers recommend using the stylet angled between 45° and 90° for optimal results (Shippey et al., 2007). Intubation with VL may occasionally be challenging, making intubation time significantly longer when compared to direct laryngoscopy (DL). In addition, insertion of the more angulated stylet loaded ETT exerts more pressure of the tissue potentially predisposing to soft tissue trauma (Walls et al., 2010; Hoshijima et al., 2018). According to Najafi et al. (2014), sore throat seems a universal issue after VL intubations with an incidence of around 30% (Najafi et al., 2014; Prekker et al., 2023).

Lee et al. sought to determine the optimal angle of the stylet when utilizing McGrath VL comparing the time to intubation. The study showed that 60° angulation was associated with shorter intubation time when compared to 90° angulation (Lee et al., 2017). Given the fact that less force is needed to achieve a grade 1 -2 view with VL, one would expect less incidence of POST and tissue trauma. However, the above-mentioned study did not look at the incidence of POST. At present, there are no data on the optimal angulation of the stylet loaded ETT which could reduce the risk of POST.

Current evidence suggests that the use of malleable stylet with steep angulation is the potential mechanism of injury and POST. Yoon et al. reported that the use of a stylet during intubation with McGrath^®^ MAC VL was associated with higer incidence of subglottic injury when compared to a group without stylet (Yoon et al., 2019). Two principal mechanisms play a role in airway trauma with intubation with a stylet loaded ETT. First, the rigid tip of stylet loaded ETT impinges on the anterior tracheal wall where it meets resistance and fails to advance resulting in subglottic injury. Second, the ETT curls anteriorly, when the stylet is removed, causing anterior subglottic trauma (Yoon et al., 2019).

Thus, the incidence of POST remains high despite the introduction of novel laryngoscopic devices and pharmacological interventions. Modifications in intubation technique allowing to address the above discussed mechanisms of injury could possibly reduce the airway trauma and, accordingly, the incidence of POST.

#### Rotation of the ETT 180° before removing the stylet (ETT 180°)

**FIGURE 1:**
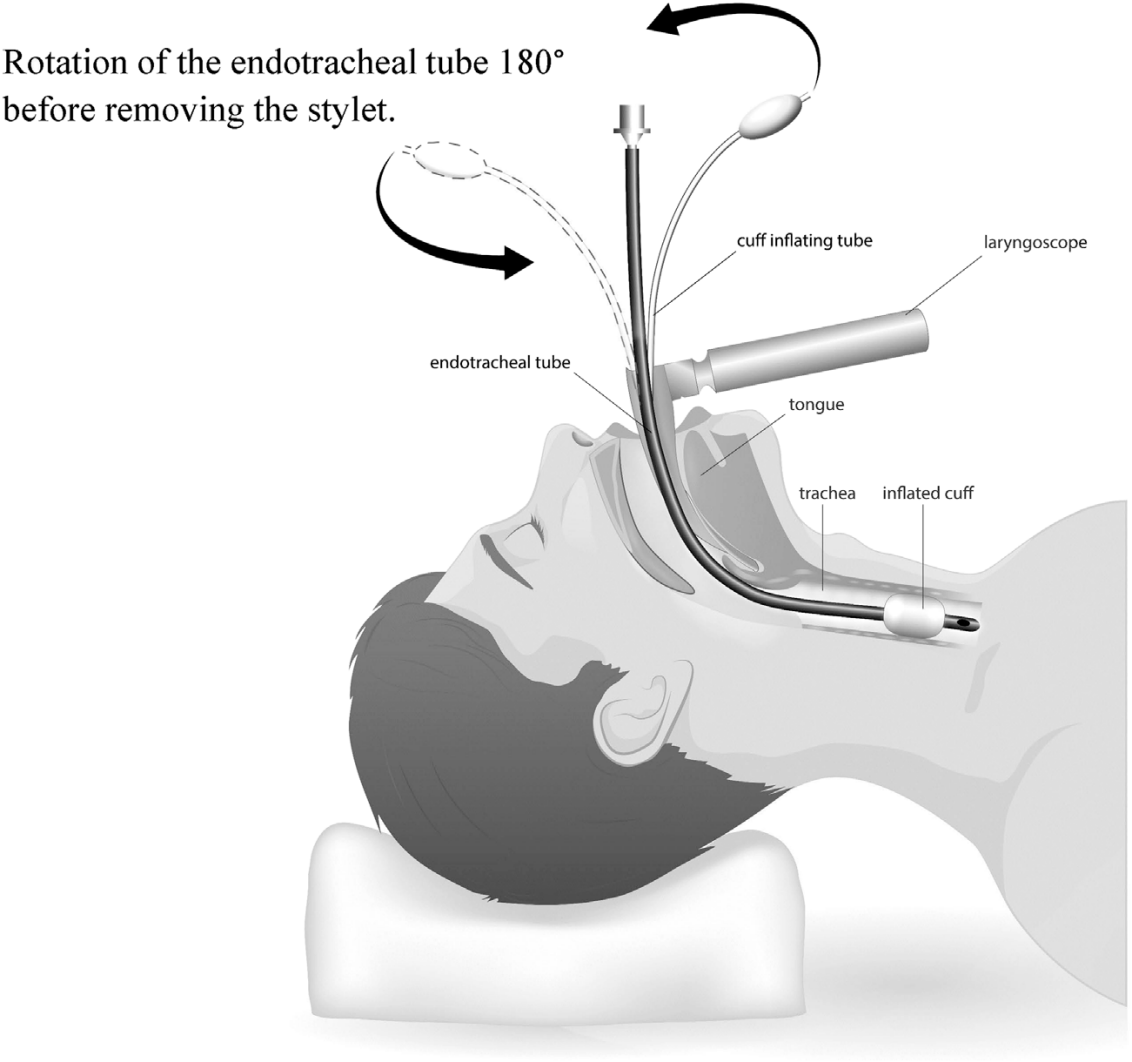
ETT 180° intubation technique.

To decrease the trauma caused by the ETT pressure on the anterior wall in the upper airway, a novel maneuver has been suggested named ETT 180°: a clockwise rotation of the stylet loaded ETT 180 degrees on its axis, once the tip of ETT passes the patient’s vocal cords (glottis) but before pulling the stylet out (Figure 1). This maneuverer allows for the stylet to match the posterior angulation of the trachea and thus minimizes the impact on the anterior wall. Once the rotation is completed, the stylet is gently removed and the ETT is advanced to the proper depth (Walls et al., 2010; Singh et al., 2013).

Unfortunately, there is scarce data regarding the incidence of POST and airway trauma related to the ETT 180° technique. In a single operator study, Seo et al. compared ETT 180° rotation of a double lumen tube (DLT) with the standard 90° rotation technique after passing the glottis. The authors reported a lower incidence of sore throat in the ETT 180 group on the first postoperative day (30/75 vs. 16/80, *p* = 0.008) and lower incidence of glottic trauma (*p* = 0.032) (Seo et al., 2013).

Additionally, this maneuver has been successfully described during selective blind endobronchial intubation in adults and children (Kubota et al., 1987).

The ETT 180° has the potential to reduce the incidence of POST and improve the quality of perioperative care. Taking this into account, our team has designed a prospective double-blinded randomized study to test the efficacy of the technique in reducing the incidence of POST. If the results of this and other studies prove the benefits of the novel technique, the ETT 180° may be introduced into clinical practice and become a new standard of care.

## Conclusion

Despite advances in laryngoscopic devices, POST remains a common problem and is associated with poor patient satisfaction requiring additional pharmacological interventions. Modifications in intubation technic taking into account the possible mechanisms of POST are required to reduce its incidence and improve that perioperative patient care.
